# Interactions Outside the Proteinase-binding Loop Contribute Significantly to the Inhibition of Activated Coagulation Factor XII by Its Canonical Inhibitor from Corn*[Fn FN2]

**DOI:** 10.1074/jbc.M114.553735

**Published:** 2014-04-04

**Authors:** Vera A. Korneeva, Mikhail M. Trubetskov, Alena V. Korshunova, Sofya V. Lushchekina, Vladimir N. Kolyadko, Olga V. Sergienko, Vladimir G. Lunin, Mikhail A. Panteleev, Fazoil I. Ataullakhanov

**Affiliations:** From the ‡ Laboratory of the Molecular Mechanisms of Hemostasis, Center for Theoretical Problems of Physicochemical Pharmacology of Russian Academy of Sciences, Moscow 119991, Russia,; the § Department of Physics, Moscow State University, Moscow 119992, Russia,; the ¶ Research Department, HemaCore LLC, Moscow 125319, Russia,; the ‖ Laboratory of Computer Modeling of Biomolecular Systems and Nanomaterials, Emanuel Institute of Biochemical Physics of Russian Academy of Sciences, Moscow 119334, Russia,; the ** Laboratory of Molecular Diagnostics and Genetic Engineering, Institute of Agricultural Biotechnology of Russian Academy of Agricultural Sciences, Moscow 127550, Russia,; the ‡‡ Laboratory of Biologically Active Nanostructures, Gamaleya Institute of Epidemiology and Microbiology of Russian Federation Ministry of Health and Social Development, Moscow 123098, Russia,; the §§ Research Division, Scientific Clinical Centre of Pediatric Hematology, Oncology, and Immunology Named after Dmitry Rogachev of Ministry of Health of Russian Federation, Moscow 117997, Russia, and; the ¶¶ Department of Translational and Regenerative Medicine, Moscow Institute of Physics and Technology, Dolgoprudny, Moscow Region, 141700, Russia

**Keywords:** Blood Coagulation Factors, Enzyme Kinetics, Enzyme Mechanisms, Protease Inhibitor, Protein Expression, Protein-Protein Interactions, Serine Protease

## Abstract

Activated factor XII (FXIIa) is selectively inhibited by corn Hageman factor inhibitor (CHFI) among other plasma proteases. CHFI is considered a canonical serine protease inhibitor that interacts with FXIIa through its protease-binding loop. Here we examined whether the protease-binding loop alone is sufficient for the selective inhibition of serine proteases or whether other regions of a canonical inhibitor are involved. Six CHFI mutants lacking different N- and C-terminal portions were generated. CHFI-234, which lacks the first and fifth disulfide bonds and 11 and 19 amino acid residues at the N and C termini, respectively, exhibited no significant changes in FXIIa inhibition (*K_i_* = 3.2 ± 0.4 nm). CHFI-123, which lacks 34 amino acid residues at the C terminus and the fourth and fifth disulfide bridges, inhibited FXIIa with a *K_i_* of 116 ± 16 nm. To exclude interactions outside the FXIIa active site, a synthetic cyclic peptide was tested. The peptide contained residues 20–45 (Protein Data Bank code 1BEA), and a C29D substitution was included to avoid unwanted disulfide bond formation between unpaired cysteines. Surprisingly, the isolated protease-binding loop failed to inhibit FXIIa but retained partial inhibition of trypsin (*K_i_* = 11.7 ± 1.2 μm) and activated factor XI (*K_i_* = 94 ± 11 μm). Full-length CHFI inhibited trypsin with a *K_i_* of 1.3 ± 0.2 nm and activated factor XI with a *K_i_* of 5.4 ± 0.2 μm. Our results suggest that the protease-binding loop is not sufficient for the interaction between FXIIa and CHFI; other regions of the inhibitor also contribute to specific inhibition.

## Teaser

**Background:** Canonical inhibitors interact with cognate proteases through a protease-binding loop.

**Results:** Protease-binding loop of corn Hageman factor inhibitor (CHFI) does not inhibit its cognate enzyme-activated coagulation factor XII (FXIIa).

**Conclusion:** Nonloop regions of CHFI are required for specific inhibition of FXIIa.

**Significance:** CHFI is the first canonical inhibitor whose protease-binding loop is not sufficient for cognate enzyme inhibition.

## Introduction

Corn Hageman factor inhibitor (CHFI)^2^ is a 14-kDa serine protease inhibitor that belongs to the I6 family, according to MEROPS (1). The mature protein consists of 127 amino acids, with a scissile bond between Arg^34^ and Leu^35^ (2). This bond is involved in interactions with both trypsin and activated coagulation factor XII (FXIIa) (2, 3). CHFI selectively inhibits FXIIa (4). FXIIa is a serine endopeptidase in the S1 family, according to MEROPS (1), and is a key enzyme of the contact pathway of plasma coagulation. However, FXIIa fails to play an important role in hemostasis *in vivo*, because patients and animals deficient in FXIIa do not suffer from bleeding disorders (5–8). Moreover, studies using experimental thrombosis (9) and ischemic stroke (10, 11) models demonstrated that mice deficient in FXIIa were resistant to experimental stress. The restoration of FXIIa to plasma concentrations led to thrombosis formation in mice.

Thus, Factor XIIa represents a promising target for antithrombotic agents because it plays no significant role in hemostasis but is involved in pathological clot formation. This scenario provides the opportunity to develop novel antithrombotic drugs and nonthrombogenic implantable medical devices (12) based on the selective inhibition of FXIIa with no risk of bleeding. Thus, interactions between FXIIa and CHFI are of great interest because of their high selectivity.

CHFI is believed to be a canonical inhibitor. Canonical inhibitors interact with their cognate proteases via a standard substrate-like mechanism (13). A convex extended protease-binding loop with a canonical conformation is responsible for inhibition (14, 15). This loop is highly complementary to the active site of the cognate protease. The scissile bond P1–P′1 (15) is located in the outermost part of the loop (16). The P1 amino acid residue is the primary determinant of the specificity of the inhibitor (13). Hydrolysis of the scissile bond does not lead to a substantial loss of inhibitory activity. Both uncleaved (*i.e.* one-chain) and modified (*i.e.* two-chain) forms of the inhibitor are active (13). The protease-binding loop of canonical inhibitors is closed, with at least one disulfide bond (17). In rare exceptions (18), this bond is replaced by strong noncovalent interactions. Although the amino acid sequences of the protease-binding loop vary greatly, inhibitory function is defined by the main chain conformation (13). Canonical inhibitors differ in folding and size, varying from 14 to ∼200 amino acids (19).

In recent decades, studies of serine protease-canonical inhibitor interactions suggested that the protease-binding loop is a minimal and sufficient base for inhibitory activity. This concept was demonstrated using both synthetic (20, 21) and recombinant (22) protease-binding loops from Bowman-Birk inhibitors. Native canonical serine protease inhibitors containing one disulfide bridge have also been described in other species, such as STFI-1 (23) from sunflower and peptides from *Odorrana grahami* (24, 25). The amphibian peptide (ORB) was further shortened to a hendecapeptide trypsin inhibitory loop that not only retained but also drastically increased its initial inhibitory activity against trypsin (*K_i_* = 306 μm for ORB and *K_i_* = 710 nm for the trypsin inhibitory loop) (26). Thus, an isolated protease-binding loop from a canonical inhibitor appears promising as a base for the design of new serine protease inhibitors.

Although the structure of the CHFI-FXIIa complex is not available, evidence suggests that CHFI is a canonical inhibitor. Both the uncleaved one-chain and cleaved two-chain forms of CHFI are reported to inhibit trypsin (27, 28) and FXIIa (3, 4). However, the two-chain form exhibits only 20–25% of the activity of the one-chain form (3, 4). The crystal structure (29) revealed that CHFI has a typical protease-binding loop that is closed via a disulfide bond and supported by an additional cysteine bridge.

Based on the available data related to small peptide serine protease inhibitors, we propose that the isolated protease-binding loop of CHFI is a promising primary structure for the development of new FXIIa inhibitors. In this study, we tested the inhibitory activity of a synthetic peptide that resembles the CHFI protease-binding loop and five recombinant truncation mutants of CHFI. Surprisingly, the cyclic peptide CHFI-2, which represents the CHFI protease-binding loop bridged with one disulfide bond, is unable to inhibit FXIIa but retains its inhibitory activity against bovine pancreatic trypsin and activated coagulation factor XI (FXIa). Our results suggest that regions outside the protease-binding loop of CHFI are likely to contribute to its inhibitory potency toward FXIIa. We also report the first simple protocol for soluble expression of CHFI in *Escherichia coli*.

## EXPERIMENTAL PROCEDURES

### 

#### 

##### Strains and Vectors

The host strain *E. coli* Rosetta-Gami 2 DE3 (EMD Millipore Corporation, Billerica, MA) was used. The expression vector pET-28a was also obtained from EMD Millipore. Recombinant CHFI and its fragments were expressed under the control of a T7 promoter and induced using isopropyl β-d-thiogalactopyranoside.

##### Primer Design, PCR Amplification, and Site-directed Mutagenesis

The pLA-TA plasmid containing a synthetic version of the CHFI gene with codon usage optimized for *E. coli* was obtained from Eurogen (Moscow, Russia). The control CHFI protein from *Zea mays* was obtained from Enzyme Research Laboratories (South Bend, IN).

The pLA-TA plasmid containing the CHFI gene was used as a PCR template for the construction of the pET28a vector containing the CHFI gene. The forward and reverse primers used in this process are as follows, with mismatches in bold type: 5′-**TGCGGATCC**TCTGCTGGTACCAGCTG-3′ and 5′-**TGCAAGCTT**AGATCTGCTCGGCATGG-3′, respectively. Specific oligonucleotides were designed to perform PCR mutagenesis for each recombinant CHFI fragment from the pET28a/CHFI template. PCR fusion was achieved as previously described (30), using forward and reverse primers and two mutagenesis primers for each mutant gene (Table 1). Vent® DNA-polymerase was obtained from New England Biolabs (Ipswich, MA). The synthetic peptide CHFI-2 was obtained from “Syneuro” (Moscow, Russia).

**TABLE 1:** **Primers for PCR mutagenesis of CHFI fragments**

	Primers (5′ → 3′)			
	Forward	Mutagenesis 1	Mutagenesis 2	Reverse
CHFI-1234	GGATCCTCTGCTGGTACCAGCTGCGTTCCGG	CAGGATAGACAGAGCGGTGTTACGGCAGTAAGCCGGGATGTC	TCCCGGCTTACTGCCGTAACACCGCTCTGTCTATCCTGATG	AAGCTTTTAGGTAGCTAAGTTGCATTCAG
CHFI-2345	GAATTCATCCCGCACAACCCGCTG	GGTGCAACGGTCGTAAGCCGGGATGTCAGCCAGTTCA	CCGGCTTACGACCGTTGCACCGCTCTG	AAGCTTAGATCTGCTCGGCATGGTACCACCA
CHFI-234	GAATTCATCCCGCACAACCCGCTG	GTGTTACGGTCGTAAGCCGGGATGTCAGCC	GCTGACATCCCGGCTTACGACCGTAACAC	AAGCTTGGTAGCTAAGTTGCATTCAGCTTC
CHFI-123	GAATTCTCTGCTGGTACCAGCTGC	GATGTCAGCCAGTTCACGGTCGCAACGACGTTTCAGTTCC	ATCCCGGCTTACTGCCGTGACACCGCTCTGTCTATCCTG	AAGCTTACCACGCTGAACTTCTCTC
CHFI-1245	GAATTCTCTGCTGGTACCAGCTGC	TCCAGAACCGACGGAATCGG	ACCGATTCCGTCGGTTCTGG	AAGCTTAGATCTGCTCGGCATGGTACCACCA

##### Restriction Cloning

The BamHI and HindIII restriction enzymes (New England Biolabs) were used to digest the obtained PCR products and the pET28a vector. T4-DNA ligase (New England Biolabs) was used to ligate the pET28a vector to each CHFI fragment gene.

##### Transformation of Host Cells

*E. coli* Rosetta-Gami 2 DE3 cells were transformed with the resulting vectors via heat shock transformation, as previously described (31), and colonies were selected on LB agar plates containing 25 μg/ml of kanamycin. Positive clones were selected by induction in 4 ml of LB containing 1 mm isopropyl β-d-thiogalactopyranoside for 2.5 h at 37 °C and confirmed by DNA sequencing.

##### Expression and Detection of Mutant Proteins

The expression and detection of CHFI mutants were accomplished by inducing the expression of *E. coli* Rosetta-Gami 2 DE3 cells harboring the appropriate plasmid. A single colony was inoculated into 4 ml of LB containing 25 μg/ml of kanamycin and grown overnight at 37 °C. A total of 1.5 liters of LB medium containing the appropriate antibiotics was inoculated with a 1:50 dilution of an overnight culture. The cells were grown at 37 °C until they reached an optical density at 600 nm of ∼0.6–0.7. Expression was induced via the addition of 0.02 mm isopropyl β-d-thiogalactopyranoside and subsequent incubation at 25 °C for 20 h. The samples were analyzed via 12% SDS-PAGE and stained with R-250 Coomassie Brilliant Blue.

##### Purification of CHFI Mutants

After overexpression of the CHFI mutant proteins, *E. coli* cells were harvested by centrifugation for 20 min at 5,000 × *g* and 4 °C. The cells were subsequently lysed in a solution of 25 mm Tris-HCl, pH 8.0, 300 mm NaCl, 1 mg/ml of lysozyme, 10 mm imidazole, and 0.1 mm PMSF with sonication. The lysates were centrifuged for 40 min at 10,000 × *g* and 4 °C. The supernatants were filtered through a 0.2-μm cellulose acetate membrane (EMD Millipore) and purified on a Ni^2+^-agarose column (Qiagen) using a gravity flow format. The column was washed with a solution of 25 mm Tris-HCl, pH 8.0, 300 mm NaCl, and 50 mm imidazole. The samples were subsequently eluted in a solution of 25 mm Tris-HCl, pH 8.0, 300 mm NaCl, and 200 mm imidazole and further analyzed via SDS-PAGE. The concentrations of the purified proteins were measured by UV absorbance at 280 nm. The extinction coefficient for each protein was calculated using Vector NTI® Advance Software (Invitrogen).

##### Inhibitory Activity Measurements

The purified proteins were diluted to reduce the imidazole concentration and concentrated on 3-kDa Millipore filters. The final protein samples were obtained in a solution of 110 mm Tris-HCl, pH 8.0, 250 mm NaCl, and 4 mm imidazole.

Chromogenic assays were performed according to the substrate manufacturer's instructions, with minor changes. Recombinant enhanced green fluorescent protein (EGFP) that was obtained under the same conditions (*i.e.* vector, host, and purification procedure) was used as a negative control. Substrates S2302 (for XIIa), S2765 (for trypsin), and S2366 (for XIa) were obtained from Chromogenix (Orangeburg, NY). Factors XIIa and XIa were obtained from Enzyme Research Laboratories and Hematologic Technologies Inc. (Essex Junction, NY), respectively. Bovine pancreatic trypsin was obtained from Sigma-Aldrich.

Activity assays for factor XIIa, factor XIa, and trypsin were performed at 37 °C in reaction buffer (110 mm Tris-HCl, pH 8.0, 250 mm NaCl, and 1% BSA) in 96-well flat-bottomed plates (Corning Inc., Corning, NY). The rate of substrate hydrolysis was recorded at 405 nm using a Sunrise microplate spectrophotometer (Tecan Group AG, Männedorf, Schweiz, Austria). We used 1 nm FXIIa and 500 μm S2302 in the FXIIa assays, 200 pm FXIa and 500 μm S2366 in the FXIa assays, and 1 nm trypsin and 500 μm S2765 in the trypsin assays. To calculate the *K_i_* values for the interaction of the CHFI mutants with FXIIa, trypsin, and FXIa, the residual activity of each protease was determined in triplicate for at least six inhibitor concentrations.

The rate of substrate hydrolysis at 405 nm (*v*) was plotted against the concentration of inhibitor on reversed coordinates to give *1*/*v* (s × OD_405_^−1^), which is dependent on *C* (nm or μm). Dependence was approximated linearly to obtain parameters *a* (*y* intercept) and *b* (slope).

The inhibitory constant *K_i_* for each experiment was calculated from the following transformed Michaelis-Menten equation,

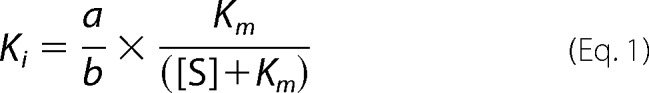
 where *K_m_* is the Michaelis constant provided by the substrate manufacturer, and [S] is the substrate concentration.

##### Statistical Analysis

Unpaired *t* tests and one-way analysis of variance were performed using GraphPad Prism software. Groups of three or more inhibitory constants representing the mean values of *n* = 2 or more measurements were compared. The *p* values are indicated on the figures.

##### Structure Preparation

The x-ray structure of CHFI-12345 at 1.95 Å resolution (Protein Data Bank (PDB) (32) code 1BEA (29)) was used. To model the CHFI-1245 mutant, Cys^29^ was manually replaced with aspartic acid. To model the CHFI-2 loop, all residues before Cys^20^ and after Cys^44^ were removed. VMD software (34) was used to prepare the model systems and further analyze the molecular dynamics (MD) trajectories (57). Missing hydrogen atoms were added, and crystallographic water molecules surrounding CHFI-12345 were included in the model. All three models of CHFI and its mutants were surrounded with TIP3P water molecules, which formed a box with boundaries at least 10 Å from the solute. Sodium and chloride ions were added to a final ion concentration of 0.15 m. NAMD 2.9 software (35) was used with a CHARMM36 force field (36). Hydrogen atoms, ions, and water molecules added to CHFI-12345, and side chains of the mutated Asp^29^ in CHFI-1245 and CHFI-2 were energy-minimized in 1,000 steps. The coordinates of atoms from the x-ray data were kept fixed. These structures of the inhibitor variants were also used in protein-protein docking and are further referred to as “model x-ray structures.”

##### Molecular Dynamics

The water box of the prepared and optimized system was equilibrated during a 1-ns MD run, with all coordinates of CHFI fixed (NPT ensemble, 298 K, 1 atm, periodical boundary conditions). The obtained model system parameters are provided in supplemental Table S1. The pre-equilibrated systems were fully energy-minimized in 5,000 steps. This step was followed by a 50-ns MD run at 298 K with parameters similar to those used in the equilibrating runs. All atoms were allowed to move. These 50-ns trajectories were used to analyze the conformational changes. To obtain stable conformations of the CHFI variants for further protein-protein docking, after the MD run at 298 K, the systems were slowly cooled to 4 K, with a temperature reduction of 1 K/1 ns. The resulting structures of the CHFI variants are further referred to as “MD structures.” The MD simulations were performed using the Lomonosov Moscow State University supercomputer (37).

##### Homology Model

Because no x-ray structure is currently available for the FXIIa catalytic domain, a homology model was built using the sequence of this domain (UniProt number P00748). The BLAST tool (38) on the UniProt web server (39) was used to find a suitable structure. The highest similarity among the proteins with available crystallographic data were observed for hepatocyte growth factor activator (UniProt number Q04756) (47% similarity). Several structures of the catalytic domain of this protein are available in the PDB, with the best resolution of 2.20 Å for structure PDB code 2R0L (40). This structure was used to build a homology model using Modeler 9v12 (41). To check the validity of the generated FXIIa homology model, we docked the tripeptide d-Pro-Phe-Arg, which is analogous to the FXIIa-specific chromogenic substrate S2302 (d-Pro-Phe-Arg-*p*-nitroaniline), in the current study. We used the protein-protein docking ClusPro server (42, 43) to estimate the initial positioning and Rosetta FlexPepDock for refinement (44, 45).

##### Protein-Protein Docking

Protein-protein docking of CHFI variants with the FXIIa homology model described above and with the x-ray structures of trypsin (PDB code 2O9Q, resolution 1.70 Å, paper in press) and FXIa (PDB code 3SOR (46), resolution 1.70 Å), was performed using the Web servers ClusPro (42, 43, 58, 59, 60) and pyDock (47, 48). For docking with pyDock, restraints (attraction) for Arg^34^ of the inhibitor and the catalytic serine were applied.

## RESULTS

### 

#### 

##### Cloning, Expression, and Purification of Recombinant CHFI and Its Mutants

**FIGURE 1.**
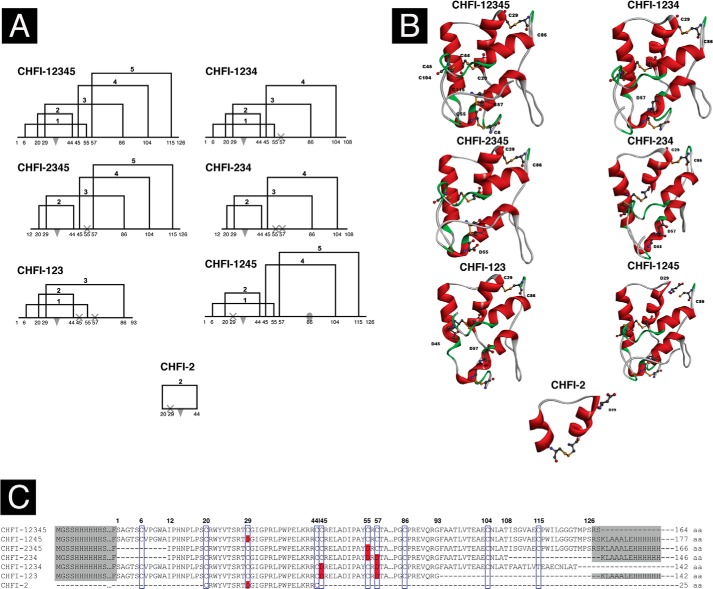
**CHFI and its mutants.** *A*, schematic diagrams of CHFI mutants. Cysteine residues and terminal amino acids are marked with *numbers* below each scheme. *Numbered square brackets* indicate disulfide bridges, *triangles* indicate the reactive site (*i.e.* the scissile bond between Arg^34^ and Leu^35^), *crosses* indicate Cys to Asp substitutions, and the *ellipse* indicates the unpaired cysteine C86. *B*, schematic diagrams of CHFI mutant conformations. Visualizations were performed using Discovery Studio software (Accelrys, Inc., San Diego, CA). α-Helices are indicated in *red*, and disulfide bridges are indicated as *yellow balls and sticks*. Cysteines and Cys to Asp mutations are labeled, and the inhibitory loop is located at the *top. C*, amino acid sequence alignment of CHFI mutants obtained using ClustalW software (38). The cysteine positions are indicated with *blue boxes*, and the positions of Cys to Asp mutations are presented in *red*. Purification tags and amino acids from the expression vector are marked with *gray shading*. The *numbers* above the sequence indicate the first and last amino acids from CHFI that are present in the mutants and the Cys residues, according to 1BEA in the PDB. The *last number* in each sequence line corresponds to the total length of the recombinant protein.

To investigate the role of CHFI regions outside the protease-binding loop in the interaction with FXIIa, a number of mutants were generated (Fig. 1) and tested. Based on the published (29) CHFI structure (PDB code 1BEA), the mutants were designed to lack different N- and/or C-terminal CHFI regions and/or disulfide bridges. CHFI-12345 is a full-size CHFI without any truncations. CHFI-2345 lacks 11 amino acids and the first disulfide bond at the N terminus, and Cys^55^ is replaced by Asp. This mutant is identical to a previously described shortened form of CHFI (49) except for the replacement of Cys^55^. Mutant CHFI-1234 lacks 24 amino acids at the C terminus and the fifth disulfide bond, with unpaired Cys^57^ replaced by Asp. Mutant CHFI-234 is a combination of the previous two truncations. This mutant lacks 11 and 24 amino acids at the N and C termini, respectively, and the first and the fifth disulfide bonds. Unpaired Cys^55^ and Cys^57^ were replaced by Asp. CHFI-123 lacks 34 amino acids at the C terminus and the fourth and the fifth disulfide bonds (Fig. 1).

To exclude interactions outside the inhibitor protease-binding loop, a cyclic 25-amino acid peptide was synthesized, with a disulfide bridge at its termini. This CHFI-2 peptide contains amino acid residues 20–45 of CHFI. Cys^29^ was replaced by Asp to avoid unwanted disulfide bond formation between unpaired cysteines. This peptide resembles the CHFI protease-binding loop. To study the effect of Cys^29^ replacement in the loop, the CHFI-1245 mutant was designed. This mutant is a full-size CHFI with Cys^29^ substituted by Asp.

DNA sequences for CHFI-12345, CHFI-1234, CHFI-2345, CHFI-234, CHFI-1245, and CHFI-123 were amplified from the synthetic CHFI gene using primers with mismatches to introduce BamHI and HindIII restriction sites and a single amino acid substitution. An additional stop codon was introduced before the HindIII restriction site in mutants CHFI-12345 and CHFI-1234; therefore, these mutants carry only an N-terminal His_6_ tag (Fig. *1**C*). After cloning into the pET28a vector, the constructs were transformed into the *E. coli* Rosetta-Gami 2 DE3 expression host.

**FIGURE 2.**
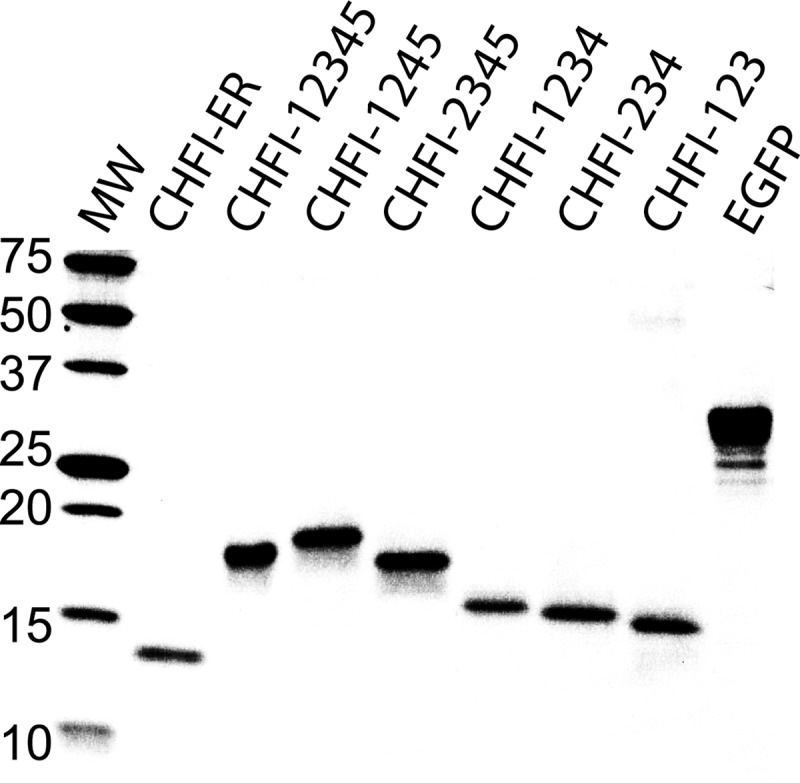
**SDS-PAGE analysis of the purity of the recombinant inhibitors.** The samples were analyzed via 12% SDS-PAGE under reducing conditions. *Lane 1* contains molecular mass standards (approximate values in kDa are presented in *lane MW*). The other lanes contain, sequentially, CHFI-ER, a control CHFI protein from *Z. mays*; recombinant CHFI-12345; mutants CHFI-1245, CHFI-2345, CHFI-1234, CHFI-234, and CHFI-123; and EGFP. Each lane contains 2 μg of protein, but 10 μg of EGFP was analyzed. The purification tags of the recombinant proteins were not removed (*i.e.* N- and/or C-terminal tag with molecular masses of 3.8 and 1.5 kDa, respectively).

The proteins were partially expressed in soluble form (data not shown), and after one-step nickel affinity purification, the samples were run on SDS-PAGE (Fig. 2). The theoretical molecular masses of the recombinant proteins with purification tags (*i.e.* N- and/or C-terminal tag with molecular masses of 3.8 and 1.5 kDa, respectively) were calculated as: 17.5 kDa for CHFI-12345, 19 kDa for CHFI-1245, 15.2 kDa for CHFI-1234, 18 kDa for CHFI-2345, 16 kDa for CHFI-234, and 15.5 kDa for CHFI-123. The theoretical molecular mass of wild type CHFI was calculated as 13.6 kDa. These values are consistent with the SDS-PAGE results (Fig. 2). The molecular mass discrepancy between control CHFI-ER from *Z. mays* seeds and recombinant CHFI (CHFI-12345) is caused by the remaining N-terminal purification tag. The approximate yields of the individual mutants ranged from 2.5 to 75 mg of purified protein/liter of cell culture. Recombinant EGFP was also expressed for further use as a negative control in chromogenic assays to exclude possible unspecific chromogenic substrate cleavage by traces of proteases from *E. coli*.

Previous attempts to obtain recombinant CHFI resulted in the preparation of nonfunctional protein because of inclusion body formation during expression in *E. coli* (49, 50). Because CHFI has five disulfide bonds (29), correct folding of the molecule requires an oxidative environment (51). However, the *E. coli* cytoplasm is slightly reducing (52). Ordinary *E. coli* strains, such as BL21 DE3 (49, 50), that were previously used for CHFI expression do not provide optimal conditions for disulfide bond formation because of the functions of the antioxidant glutathione and thioredoxin systems (52). Based on these observations, the *E. coli* Rosetta-Gami 2 DE3 strain with mutations in the glutathione and thioredoxin systems (53) was chosen as a host for the production of CHFI and its mutants. Thus, the observed soluble expression of CHFI is consistent with the data obtained in proof of method studies (54).

##### Inhibitory Activity Assay

**FIGURE 3.**
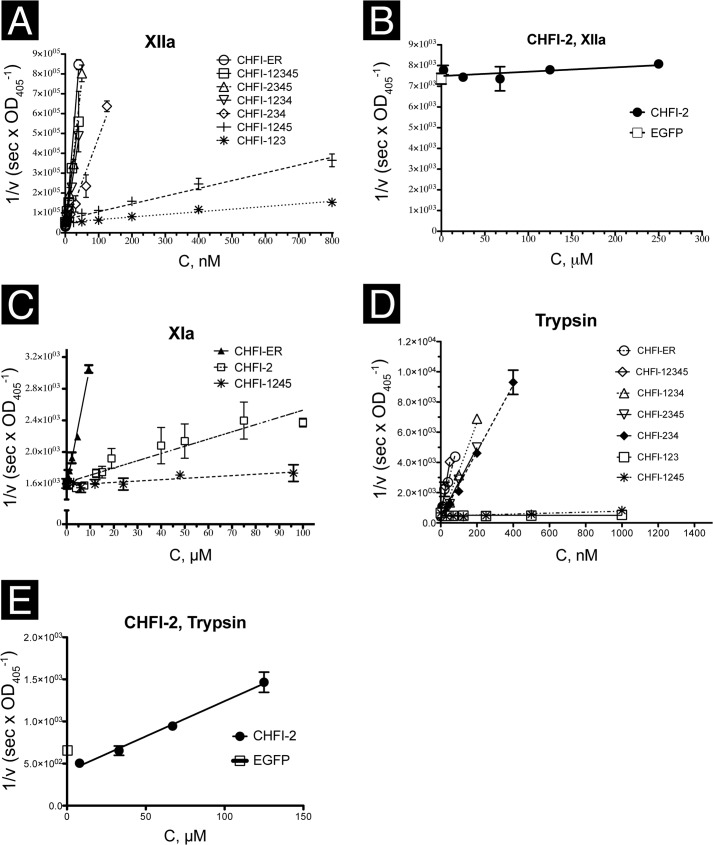
**Inhibition of trypsin, FXIIa, and FXIa activity by CHFI mutants.** *A–E*, the rate of substrate cleavage by the corresponding protease in reversed coordinates (1/*v*) on the ordinate axis and depending on the inhibitor concentrations (*abscissa* axis) obtained in the chromogenic assays. The mean values ± S.E. are plotted. Recombinant EGFP that was prepared under the same experimental conditions as recombinant CHFI was used as a negative control. *A*, inhibition of FXIIa cleavage of S2302 by native CHFI (CHFI-ER), recombinant CHFI (CHFI-12345), and the mutant variants. *B*, inhibition of FXIIa cleavage of S2302 cleavage by the synthetic cyclic peptide CHFI-2. The slope is not significant. *C*, inhibition of FXIa cleavage of S2366 cleavage by native CHFI (CHFI-ER), its mutant CHFI-1245, and the synthetic cyclic peptide CHFI-2. *D*, inhibition of trypsin cleavage of S2765 by recombinant CHFI (CHFI-12345) and its mutant variants. *E*, inhibition of trypsin cleavage of S2765 by the synthetic cyclic peptide CHFI-2.

To investigate inhibitory activity against FXIIa, FXIa, and trypsin, the purified mutant proteins were tested in chromogenic assays (Fig. 3 and Tables 2 and 3).

**TABLE 2:** **Comparison of the *K_i_* values of the mutants against XIIa factor and trypsin** ND, not determined.

Inhibitor	*K_i_*	
	XIIa	Trypsin
	*nm*	
CHFI-ER	1.0 ± 0.1	2.1 ± 0.6
CHFI-12345	1.1 ± 0.2	1.3 ± 0.2
CHFI-2345	1.0 ± 0.2	2.9 ± 0.5
CHFI-1234	1.0 ± 0.3	1.0 ± 0.6
CHFI-234	3.2 ± 0.4	2.2 ± 1.1
CHFI-1245	50 ± 8	250 ± 20
CHFI-123	116 ± 16	ND*^a^*
CHFI-2	ND*^b^*	11,700 ± 1200

*^a^* No inhibition was observed for CHFI-2 at concentrations up to 1 mm (data not shown).

*^b^* No inhibition was observed for CHFI-123 at concentrations up to 75 μm (data not shown).

**TABLE 3:** **Comparison of the *K_i_* values of wild-type CHFI-ER, CHFI-2, and CHFI-1245 against factor XIa**

Inhibitor	*K_i_* XIa
	μ*m*
CHFI-ER	5.4 ± 0.2
CHFI-2	94 ± 11
CHFI-1245	490 ± 110

##### FXIIa

**FIGURE 4.**
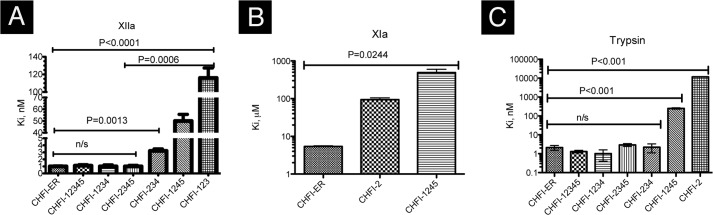
**Comparison between the inhibition constants (*K_i_*) of recombinant CHFI (CHFI-12345) and its mutant variants.** *A–C*, *K_i_* values of recombinant (CHFI-12345) CHFI and its mutant variants against FXIIa (*A*), FXIa (*B*), and trypsin (*C*). *n/s*,5 not significant.

The function of recombinant CHFI is indistinguishable from that of wild-type CHFI (CHFI-ER) from corn seeds. This finding suggests that the remaining purification tags do not influence the inhibitory activity of CHFI against FXIIa. Shortened mutants with the truncation of only one disulfide bond at the N (*i.e.* CHFI-2345) or C terminus (*i.e.* CHFI-1234) also inhibit FXIIa with the same activity as wild-type CHFI. The truncation of one disulfide bond from both the N terminus and the C terminus (*i.e.* CHFI-234) slightly decreases the efficiency of the inhibitor (*K_i_* = 3.2 ± 0.4 nm *versus K_i_* = 1.0 ± 0.1 nm for wild type; *p* value < 0.05) (Fig. 4*A*). The truncation of the fourth and fifth disulfide bonds (*i.e.* CHFI-123) dramatically reduces the ability of the mutant to inhibit FXIIa (*K_i_* = 116 ± 16 nm) compared with the wild-type protein (*p* value < 0.001). A full-size CHFI mutant lacking the third disulfide bond also exhibits reduced inhibitory activity against FXIIa (*K_i_* = 50 ± 8 nm, *p* value < 0.05) (Fig. 4*B*). Inhibition of FXIIa by the synthetic peptide CHFI-2, which represents the protease-binding loop, was not observed (Fig. 3*B* and Table 2) for peptide concentrations up to 1 mm (data not shown). Thus, the mutants retain wild-type inhibitory activity until the third and fourth disulfide bonds are deleted from the molecule, with a drastic decrease observed for CHFI-123.

##### Trypsin

Because no FXIIa inhibition by CHFI-2 was observed, this peptide and the other mutants were assayed against trypsin (Fig. 3, *D* and *E*, and Table 2). The observed inhibitory activity of the purified mutant proteins is similar with respect to the level of magnitude and varies nonsignificantly (Figs. 3, *D* and *E*, and 4*C*) from 1 ± 0.6 nm for CHFI-1234 to 2.9 ± 0.5 nm for CHFI-2345, as long as disulfide bridges 2, 3, and 4 are present. CHFI-1245 exhibited a dramatically increased *K_i_* (250 ± 70 nm), as did the synthetic peptide CHFI-2 (12 ± 2 μm). No inhibitory activity was observed for CHFI-123 at concentrations up to 75 μm (data not shown). Because CHFI-2 retains partial inhibitory activity, the isolated protease-binding loop might be in the active conformation.

##### FXIa

To determine whether the inhibitors with decreased activity exhibited weak interactions with the cognate enzyme, CHFI-2 and CHFI-1245, which serves as a control for the active loop conformation, were assayed against FXIa. CHFI-2 retains partial inhibitory activity against FXIa, with *K_i_* = 94 ± 11 μm, whereas wild-type CHFI inhibited FXIa with *K_i_* = 5.4 ± 0.2 μm. Unexpectedly, CHFI1245 lost the ability to inhibit FXIa (Fig. 3*C* and Table 3).

##### Modeling of the Interactions between the Isolated Protease-binding Loop CHFI-2 and Cognate Enzymes

To analyze the possible causes of CHFI-2 inactivity against FXIIa, protein-protein docking was performed. Because the x-ray structure of the complete FXIIa molecule is not currently available, the FXIIa structure was modeled prior to the docking procedure.

##### Molecular Dynamics

To investigate whether the mutations cause conformational changes in the isolated protease-binding loop (CHFI-2) and CHFI-1245 (*i.e.* CHFI-12345 with a C29D mutation), MD simulations were performed. A 50-ns MD simulation was followed by cooling of the system to obtain conformations of all three inhibitor variants stable in solution. These conformations might differ from the conformation of CHFI-12345 in the crystalline phase that was provided by the x-ray data. The conformation of the Arg^34^ side chain, which is a residue located at the scissile bond, is of particular interest.

**FIGURE 5.**
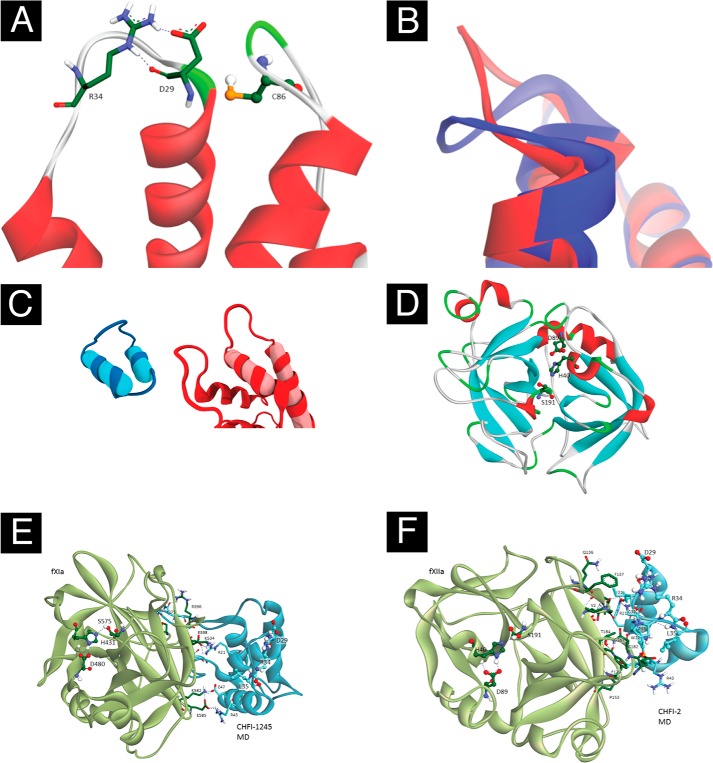
**CHFI mutant protein-binding loop conformation, FXIIa model structure, and docked FXIa-CHFI-1245 and FXIIa-CHFI-2 complexes.** *A* and *B*, conformation of the loop carrying Arg^34^ in CHFI-1245, as indicated by the molecular dynamics simulation. The salt bridge between Arg^34^ and Asp^29^ (*A*) is shown with *dashes*. An overlap of the CHFI-12345 (*blue*) and CHFI-1245 (*red*) structures after MD simulation is presented in *B. C*, the angle between α-helixes in CHFI-2 (*left*) differs from that of CHFI-12345 (*right*), as indicated by the MD simulation. The axes of α-helixes are shown as *cylinders*. The structures are visualized using the Bendix plugin (56) for VMD. *D*, model structure of FXIIa, where catalytic residues are shown as *balls and sticks. E* and *F*, protein-protein docking of protease complexes with inhibitor, which is bound tightly to the target far from the active site: FXIa and CHFI-1245 (*E*) and FXIIa and CHFI-2 (*F*).

During the MD simulation of CHFI-12345, the C29D mutant CHFI-1245, and the synthetic cyclic peptide CHFI-2, no dramatic conformational changes were observed. After cooling the CHFI-12345 system, the conformation of the Arg^34^ side chain, which is one of the amino acids from the scissile bond Arg^34^–Leu^35^, differed from the initial x-ray conformation. In CHFI-1245 and CHFI-2, Arg^34^ is similarly bent down to the backbone. However, in CHFI-1245 (*i.e.* the C29D mutant), Arg^34^ forms a stable salt bridge with mutated Asp^29^ (Fig. 5*A*), causing a change in the shape of the loop carrying Arg^34^ (Fig. 5*B*). In CHFI-2, the angle between two α-helixes is different from that observed in the initial x-ray structure (Fig. 5*C*). Because of this conformational change, the structure of the cyclic peptide is more rigid; as a result, Arg^34^ and Asp^29^ are unable to form a salt bridge because they are separated by a distance of 7 Å, which is sufficient to dampen their interaction. These differences might alter the interactions of the CHFI variants with the active sites of their cognate enzymes. Because of the observed difference in the conformation of the Arg^34^ side chain between the x-ray structure and the structure obtained after MD cooling, two forms of each inhibitor were used for protein-protein docking: the x-ray structures with minimization of modified parts and the stabilized geometries after 50-ns MD at 298 K and slow cooling of the system to 4 K. A detailed account of the molecular modeling results is presented in the supplemental materials.

##### Protein-Protein Docking

Homology modeling of the FXIIa structure provided a model with a serine-histidine-aspartic acid triad located in a cleft on the protein surface in a suitable position for performing catalytic substrate cleavage (Fig. 5*D*). Docking of the tripeptide analog of the FXIIa-specific substrate S2302 provided a position in the model active site that was suitable for peptide bond cleavage (see SI). Thus, this FXIIa model was used for protein-protein docking without further improvements. A detailed description of the protein-protein docking results for the three inhibitor variants and their cognate enzymes is provided in the SI.

In most cases, more favorable scoring energies of protein-inhibitor complexes were obtained for conformations of CHFI and its analogs that were provided by comparing the MD simulations to the x-ray structure and its modifications (*i.e.* the C29D mutant and the truncated cyclic fragment) (supplemental Table S2). This finding indicates that conformations obtained after cooling the system in the MD simulation (*i.e.* stable in solution) for the inhibitor variants are more suitable for inhibition of the target enzymes than the crystalline phase conformation provided by the x-ray data (see “Molecular Dynamics” in the supplemental materials for details). The protein-protein docking results for these MD structures are consistent with the experimental observations of inhibitory activity described above. All three CHFI variants exhibit good binding with the trypsin active site in a position favorable for cleavage of the bond between Arg^34^ and Leu^35^, but the binding of the cyclic peptide is weaker than that of the full-length inhibitors. This finding is consistent with the data obtained from trypsin inhibition assay (Table 2 and Fig. 3, *D* and *E*). The docked position of CHFI-12345 with FXIa places Arg^34^ in the active site, but the position is relatively far from the catalytic serine. In addition, the scoring energy of the binding is rather poor compared with the energies obtained for the complexes with trypsin. Taken together, these data could explain the experimentally measured 3-fold increase in inhibition constants. In the case of CHFI-1245, the observed position is rather far from the active site and is stabilized by several salt bridges (Fig. 5*E*). The existence of such an alternative binding site could explain the poor experimentally observed inhibitory activity of CHFI-1245 against FXIa (Table 3 and Fig. 3*C*). The interaction of the cyclic synthetic peptide CHFI-2 with FXIa is similar to the binding of CHFI-2 to trypsin. The complexes between the FXIIa model and CHFI-12345 and its C29D mutant were similar to those of trypsin. However, in the case of CHFI-2, the most favorable position is located far from the active site and generated primarily by hydrophobic contacts (Fig. 5*F*).

## DISCUSSION

Here we report that interactions outside the protease-binding loop of CHFI and the active site of FXIIa are likely to contribute to the strength and specificity of binding. This finding suggests that CHFI is likely the first canonical inhibitor whose protease-binding loop is not sufficient for cognate enzyme inhibition. According to our results, the synthetic cyclic peptide CHFI-2 lacks inhibitory activity against FXIIa. Moreover, FXIIa-CHFI-2 docking results suggest that the peptide is likely to bind far from the active site of FXIIa. This finding is not expected for a canonical inhibitor because an isolated protease-binding loop is reported to be an independent structural element (20, 21) and to determine interactions with the cognate enzyme. For instance, a cyclic peptide resembling the loop from SFTI-1 retains the inhibitory activity and structural features of the full-size inhibitor and has a *K_i_* (19 nm) ∼40 times higher than that of the parental protein (0.5 nm) (20).

**FIGURE 6.**
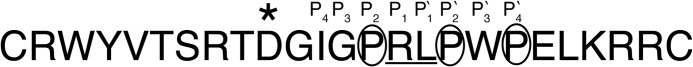
**The amino acid sequence of CHFI-2.** The loop is rich in prolines (indicated with *circles*). The *asterisk* indicates the replacement of Cys^29^ with Asp. The scissile bond is *underlined*, and the amino acid positions relative to the reactive site are indicated.

CHFI-2 (*i.e.* the loop) is rich in proline (Figs. 1*C* and 6) and should therefore have a stable conformation. We initially hypothesized that despite the presence of prolines, the isolated loop might become more flexible than the corresponding region of the original protein. Such conformational destabilization could affect tight interactions with FXIIa while exerting less influence on weak interactions with FXIa. Therefore, CHFI-2 inhibitory activity was assayed for proteases that are specifically inhibited by CHFI (*i.e.* trypsin and FXIIa) and for FXIa, for which the inhibition is much weaker. MD simulation and protein-protein docking were also performed to predict possible interactions between the peptide and its cognate enzymes.

The MD results suggest that the spatial structure of the cyclic peptide is quite rigid, and no dramatic conformational changes compared with the loop in the wild-type inhibitor are proposed (see Section 1.3, “CHFI-2: The Cyclic Synthetic Peptide,” in the supplemental materials for details). Only slight alterations of the angle between two α-helixes in CHFI-2 were observed compared with the initial x-ray structure of the loop (Fig. 5*C*). These results do not support the initial assumption that the isolated loop would exhibit extra flexibility.

Our experimental results from the inhibition assay for the CHFI-2 peptide are consistent with previous findings (20, 21). These results suggest that the isolated protease-binding loop retains partial inhibitory activity and acts as an independent structural element. CHFI-2 retains inhibitory activity against FXIa, but the *K_i_* for this interaction (94 ± 11 μm) is ∼20 times higher than that of wild-type CHFI (5.4 ± 0.2 μm). CHFI-2 also retains its inhibitory activity against trypsin; however, the difference in *K_i_* between CHFI-2 and wild-type CHFI is greater (12 ± 2 μm *versus* 1.3 ± 0.2 nm, respectively). Taken together with the MD results, these data suggest that the isolated protease-binding loop is likely to be in an active conformation because it can inhibit trypsin and FXIa. The docking results support these experimental data, because the scissile bond of the peptide (*i.e.* Arg^34^–Leu^35^) is located in the active site of trypsin and FXIa.

Surprisingly, no inhibition was observed when CHFI-2 was assayed against FXIIa (Fig. 3*B*). Because the peptide is supposed to be in the active conformation, this lack of inhibition could be explained by an incorrect position of the scissile bond relative to the FXIIa catalytic site. We hypothesize that the functional position of the CHFI protease-binding loop in the cave of the FXIIa catalytic site is primarily determined by interactions between nonloop regions of CHFI and FXIIa. This hypothesis is supported by the CHFI-2-FXIIa docking results. The most favorable calculated position of the peptide is outside of the predicted active site on the FXIIa model structure (Fig. 5*F*). However, the disproportional loss of CHFI-2 inhibitory activity against FXIIa compared with trypsin and FXIa could also be partially caused by the elimination of the third disulfide bond and the other interactions between the loop and nonloop regions of CHFI.

To better understand these possible reasons for the lack of activity of the CHFI-2 peptide against FXIIa, the role of CHFI protease-binding loop stabilization by the third disulfide bond was studied in experiments with CHFI-1245, which is a full-size CHFI mutant with the third disulfide bridge eliminated. This mutant was also used as a control for the C29D replacement in the CHFI-2 peptide. CHFI-1245 exhibits reduced inhibitory activity against FXIIa and trypsin (*K_i_* = 50 ± 8 and 250 ± 20 nm, respectively) compared with wild-type CHFI (*K_i_* = 1.0 ± 0.1 and 1.3 ± 0.2 nm, respectively). Unexpectedly, CHFI-1245 lost the ability to inhibit FXIa (*K_i_* = 490 ± 110 μm). This finding could be explained by the CHFI-1245-FXIa docking results, because an alternative CHFI-1245 binding site was present on the FXIa surface outside the active site (Fig. 5*E*). Nevertheless, the residual inhibitory activity of CHFI-1245 against FXIIa and trypsin suggests that although the third disulfide bridge contributes to the interaction of the CHFI loop with the FXIIa active site, the C29D substitution is not likely to be the only cause of the lack of activity of the CHFI-2 peptide against FXIIa.

Our further studies of the contact region in the interaction between CHFI and FXIIa suggest that either a region between CHFI residues Gly^93^ and Thr^108^ interacts with FXIIa or the fourth disulfide bond is essential for proper folding of CHFI. The elimination of the first and the fifth disulfide bonds does not affect the folding of the contact region of the truncated protein with FXIIa. Considering the conformation-based concept of the structure-activity investigation (55) and the observation (49) that a mutant that lacks 11 N-terminal amino acids and has a Cys^55^ substitution is indistinguishable from full-size recombinant CHFI, we investigated the inhibitory activity of four truncated CHFI mutants in comparison with the wild-type and full-size recombinant CHFI (CHFI-12345). The shortened mutants CHFI-1234 (*K_i_* = 1.0 ± 0.3 nm for FXIIa) and CHFI-2345 (*K_i_* = 1.0 ± 0.2 nm) retain the ability to inhibit FXIIa to the same extent as wild-type CHFI (*K_i_* = 1.0 ± 0.1 nm); this finding is consistent with previously reported results (49). CHFI-234 is a combination of the truncations from CHFI-1234 and CHFI-2345. This mutant has a slightly increased *K_i_* against FXIIa (3.2 ± 0.4 nm), which is similar to of CHFI-1234, CHFI-12345, and CHFI-2345 at a 0.01 level of significance (*p* value = 0.0013, which is <0.01; Figs. 3*A* and 4*A*). Similar results were obtained in the trypsin inhibition assay (Table 2 and Figs. 3, *D* and *E*, and 4*C*). Together, these results suggest that the 11 N-terminal and 24 C-terminal amino acids of CHFI and the first and the fifth disulfide bonds are not involved in interactions with FXIIa and trypsin. Therefore, CHFI-234 is a minimally active CHFI fragment that is necessary for FXIIa inhibition, and CHFI-2 is an independent structural element that retains its inhibitory activity against trypsin and FXIa.

Interactions outside the FXIIa active site and the CHFI protease-binding loop contribute to the strength and the specificity of binding, because the isolated binding loop is unable to inhibit FXIIa but partially retains inhibitory activity against other proteases, such as trypsin and FXIa. These findings suggest that the mechanism of FXIIa inhibition by CHFI could deviate from the standard mechanism (13), with nonloop regions involved in the process in an unknown manner. In contrast, our results indicate that CHFI behaves according to the standard mechanism of inhibition, in which the protease-binding loop plays a crucial role, during the inhibition of trypsin and FXIa.

## Footnotes

2 The abbreviations used are:

CHFI: corn Hageman factor inhibitor
FXIIa: activated coagulation factor XII
FXIa: activated coagulation factor XI
EGFP: enhanced green fluorescent protein
PDB: Protein Data Bank
MD: molecular dynamics.
